# Mendelian randomization analysis identified tumor necrosis factor as being associated with severe COVID-19

**DOI:** 10.3389/fphar.2023.1171404

**Published:** 2023-06-16

**Authors:** Hongfei Song, Na Lei, Ling Zeng, Xiuyan Li, Cen Jiang, Quansheng Feng, Yue Su, Jibin Liu, Jie Mu

**Affiliations:** Traditional Chinese Medicine and Inflammation Regulation Research Group, School of Basic Medical Sciences, Chengdu University of Traditional Chinese Medicine, Chengdu, China

**Keywords:** COVID-19, genome-wide association study, tumor necrosis factor, Mendelian randomization analyses, causal associations

## Abstract

**Background:** Observational studies have shown that anti-tumor necrosis factor (TNF) therapy may be beneficial for patients with coronavirus disease 2019 (COVID-19). Nevertheless, because of the methodological restrictions of traditional observational studies, it is a challenge to make causal inferences. This study involved a two-sample Mendelian randomization analysis to investigate the causal link between nine TNFs and COVID-19 severity using publicly released genome-wide association study summary statistics.

**Methods:** Summary statistics for nine TNFs (21,758 cases) were obtained from a large-scale genome-wide association study. Correlation data between single-nucleotide polymorphisms and severe COVID-19 (18,152 cases vs. 1,145,546 controls) were collected from the COVID-19 host genetics initiative. The causal estimate was calculated by inverse variance-weighted (IVW), MR–Egger, and weighted median methods. Sensitivity tests were conducted to assess the validity of the causal relationship.

**Results:** Genetically predicted TNF receptor superfamily member 6 (FAS) positively correlated with the severity of COVID-19 (IVW, odds ratio = 1.10, 95% confidence interval = 1.01–1.19, *p* = 0.026), whereas TNF receptor superfamily member 5 (CD40) was protective against severe COVID-19 (IVW, odds ratio = 0.92, 95% confidence interval = 0.87–0.97, *p* = 0.002).

**Conclusion:** Genetic evidence from this study supports that the increased expression of FAS is associated with the risk of severe COVID-19 and that CD40 may have a potential protective effect against COVID-19.

## 1 Introduction

Coronavirus disease 2019 (COVID-19) is caused by the infection of the respiratory system with severe acute respiratory syndrome coronavirus 2 (SARS-CoV-2), a member of the betacoronavirus family. According to a survey from the Center for Systems Science and Engineering at Johns Hopkins University, as of 3 March 2023, there were over 676 million confirmed cases of COVID-19 worldwide, including over 6.88 million fatalities (CSSE, 2023). Several treatments have been investigated during the pandemic, including antiviral drugs, anti-inflammatory drugs, cellular therapy, plasma therapy, monoclonal antibodies, and intravenous immunoglobulin therapy (Moeinafshar et al., 2022; Niknam et al., 2022; Lin et al., 2023; Yang et al., 2023). However, some of them were revealed to have no therapeutic effect on COVID-19 (Abdool Karim and Devnarain, 2022; Marcec et al., 2022).

Many studies have demonstrated that an excessive inflammatory response may be a key contributor to an adverse outcome of COVID-19 (Heidari Nia et al., 2022; Zheng et al., 2022; Falck-Jones et al., 2023; Li et al., 2023) and that tumor necrosis factors (TNFs) play a vital role in initiating the inflammatory cascade response. Furthermore, observational studies have indicated that TNF therapy may be beneficial for patients with COVID-19 (Evangelatos et al., 2022; Tripathi et al., 2022). Additionally, a retrospective study by Stallmach et al. (2020) found a decline in pro-inflammatory cytokines in seven patients with severe COVID-19 but no potential immune-mediated inflammatory disease who received a single dose of infliximab 5 mg/kg within 0–3 days of admission. Only one of the seven patients, with a median age of 60 years, died. Nonetheless, whether TNFs are genetically causally associated with severe COVID-19 remains largely unclear because of possible confounding factors and reverse causality in traditional observational studies. Therefore, this study extracted large genome-wide association study (GWAS) data for TNFs for two-sample Mendelian randomization (MR) analysis to resolve this issue.

MR utilizes genetic variation to identify whether the observed correlation between risk factors and outcomes is concordant with a causal effect (Smith and Ebrahim, 2003). In general, the use of MR analysis minimizes confounding bias and avoids reverse causality. In the absence of large randomized controlled trials, MR methods are an important alternative strategy to provide credible evidence for a causal relationship between exposure and disease risk (Zuccolo and Holmes, 2017). Currently, MR analysis has an essential role in predicting disease risk factors, especially during pandemics (Baranova et al., 2022; Chen et al., 2022; Au Yeung et al., 2023). For the work described herein, MR was applied to assess the potential causal contribution of nine TNFs to severe COVID-19.

## 2 Methods

A two-sample MR analysis was performed to examine the causal effects of nine TNFs on COVID-19 severity using GWAS pooled statistics. This MR study was based on three important assumptions: i) genetic instruments predict the exposure of interest (*P* < 5E-06); ii) genetic instruments are independent of potential confounders; and iii) genetic instruments affect the outcome through risk factors only (Davies et al., 2018).

### 2.1 Data source

A recent study by Folkersen et al. (2020) mapped and replicated protein quantitative trait loci (pQTLs) for 90 cardiovascular proteins in more than 30,000 participants. A total of 90 proteins from 21,758 individuals within 13 European pedigree cohorts passed quality control criteria for use in GWAS meta-analysis. This study determined and duplicated 315 primary and 136 secondary pQTLs for 85 of these cyclins, providing new insights for translational research and drug development. The pQTL-based framework was designed to solve several critical tasks related to drug development, which included (a) mapping of protein regulatory pathways, (b) identification of new target candidates, (c) drug repositioning, (d) target-related safety, and (e) matching target mechanisms to patients through protein biomarkers or genetic polygenic risk scores (Folkersen et al., 2020). The findings provided a comprehensive toolbox for evaluating and developing therapeutic hypotheses and precision medicine methods for complex diseases. Supplementary Table S1 lists the details of the nine TNF-related phenotypes involved in our study. These nine TNFs are as follows: TNF-related activation-induced cytokine (TRANCE); TNF-related apoptosis-inducing ligand (TRAIL); TNF-related apoptosis-inducing ligand receptor 2 (TRAIL-R2); tumor necrosis factor ligand superfamily member 14 (TNFSF14); tumor necrosis factor receptor 1 (TNF-R1); tumor necrosis factor receptor 2 (TNF-R2); tumor necrosis factor receptor superfamily member 5 (CD40); tumor necrosis factor receptor superfamily member 6 (FAS); and CD40 ligand (CD40L). The genome-wide significance of TNF-related single-nucleotide polymorphisms (SNPs) (*P* < 5E-06) was used as an instrumental variable.

**FIGURE 1:**
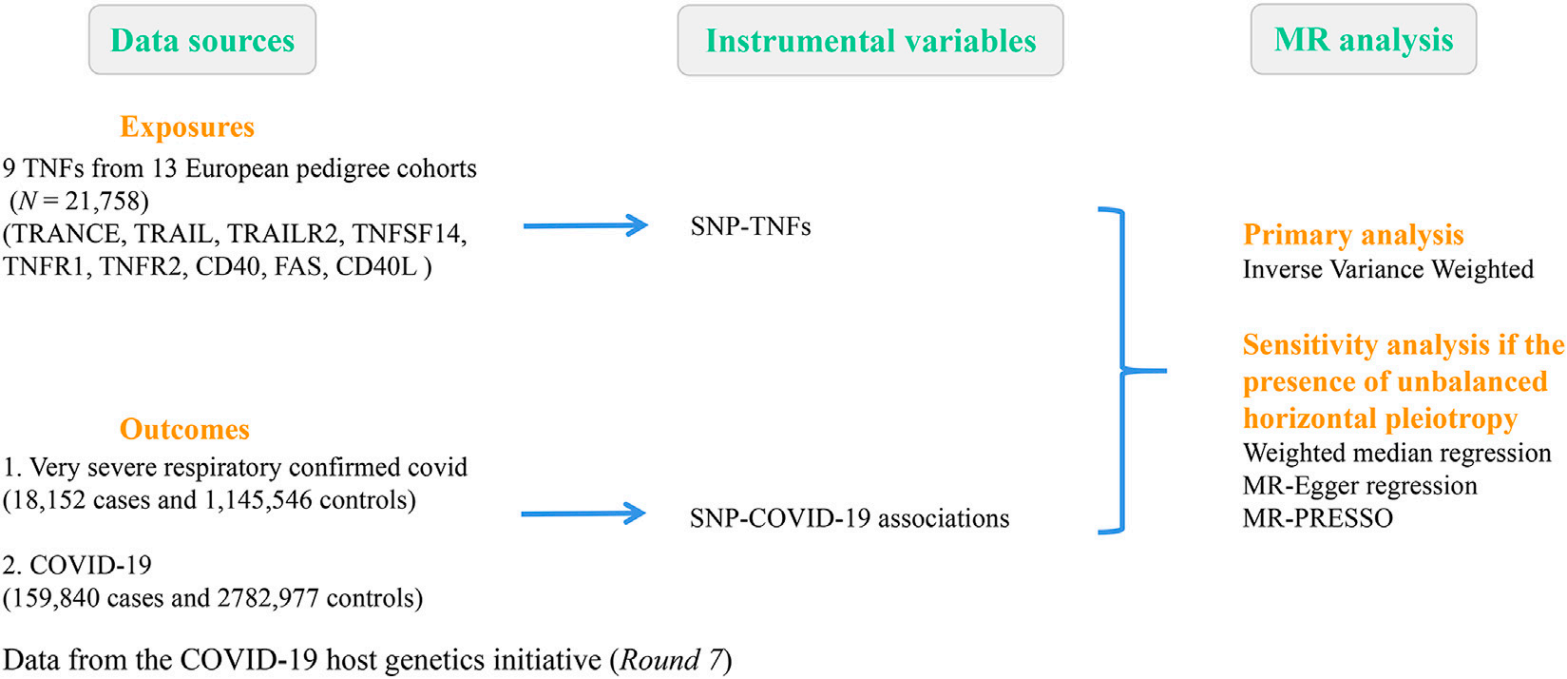
Summary of data sources and flowchart of the research design.

TRANCE, TNF-related activation-induced cytokine; TRAIL, TNF-related apoptosis-inducing ligand; TRAIL-R2, TNF-related apoptosis-inducing ligand receptor 2; TNFSF14; tumor necrosis factor ligand superfamily member 14; TNF-R1, tumor necrosis factor receptor 1; TNF-R2, tumor necrosis factor receptor 2; CD40, tumor necrosis factor receptor superfamily member 5; FAS, tumor necrosis factor receptor superfamily member 6; and CD40L, CD40 ligand.

The COVID-19 host genetics initiative was an international genetics collaboration that intended to elucidate the genetic determinants of COVID-19 susceptibility and severity-related outcomes (The COVID-19 Host Genetics Initiative, 2020). To achieve this goal, researchers worldwide collected individual-level clinical and genetic data and carried out GWAS analyses. This study selected summary statistics from GWAS meta-analysis *Round 7* involving 18,152 patients with confirmed very severe respiratory COVID-19 released on 3 April 2022 (https://www.covid19hg.org/results/r7/). Details of the GWAS dataset for severe COVID-19 included within our work are shown in Figure 1 and Supplementary Table S1.

### 2.2 Statistical analyses

This study first harmonized effect alleles for exposures and outcomes and then used inverse variance-weighted (IVW), weighted median, and MR‒Egger analyses to determine MR estimates of exposure *versus* outcome. IVW is most widely used in MR studies and was considered the primary outcome of our study because of its ability to provide reliable causal estimates without directional pleiotropy (Bowden et al., 2015). Weighted median and MR‒Egger methods are utilized to complement IVW estimates because they present more reliable estimates over a wider range of situations but are less efficient. The linkage disequilibrium threshold was set to *r* ^2^ < 0.01 within a clumping window of 1,000 kb.

The listed SNPs were excluded in the following cases. First, no specific requested SNPs existed during the extraction of specific SNPs from the resulting GWAS, and it was not possible to search for proxies with requested SNPs in LD from the resulting GWAS. Second, the effect of ambiguous SNPs with incongruent alleles or palindromic SNPs with ambiguous strands could not be corrected. In addition, to assess the strength of each instrumental variable, the F-statistic (F-statistic≥10 suggested that the selected SNPs were valid with sufficient strength) was calculated for each SNP (Burgess et al., 2011). *R* ^2^ and F-statistics were computed as follows:

$$R^{2}=2\times \left(1-EAF\right)\times EAF\times \beta^{2},$$

$$F=\left(\frac{R^{2}}{1-R^{2}}\right)\left(\frac{N-k-1}{k}\right).$$

β, genetic estimate of exposure per SNP; N, sample size (number of cases plus controls); k, number of SNPs; EAF, effect allele frequency; and SE, standard error.

Sensitivity tests were conducted to ensure the stability of the results. First, Cochran’s Q test was conducted to assess heterogeneity in individual causal effects (a value of *p* > 0.05 was considered to indicate no heterogeneity). Then, MR‒Egger regression was used to evaluate the horizontal pleiotropy of the instruments (a value of *p* > 0.05 was considered to indicate no pleiotropy). If horizontal pleiotropy was found, the MR-pleiotropy residual sum and outlier methods (MR-PRESSO) were performed to correct for horizontal pleiotropy. Finally, a leave-one-out test was performed to evaluate the effect of individual variation on the observed associations.

The results are expressed as odds ratios (ORs) and their 95% confidence intervals (CIs). Statistical significance was accepted for *p* < 0.05. All analyses were carried out with R statistical software (version 4.2.1) with the R packages “TwoSampleMR” and “MRPRESSO.”

## 3 Results

### 3.1 Identification of genetic instruments

For each of the 9 TNFs (TRANCE, TRAIL, TRAIL-R2, TNFSF14, TNF-R1, TNF-R2, CD40, IRF, FAS, and CD40L), SNPs exceeding the genome-wide significant *p*-value (*P* < 5E-06) were selected as genetic instruments. The number of genetic instruments varied from 14 for TRAIL-R2 to 26 for TNF-R2 (Supplementary Table S2). The F-statistics of each SNP varied from 15.54 to 1864.79, indicating a low probability of weak instruments (Pierce et al., 2011).

### 3.2 Associations of TNFs with severe COVID-19

**FIGURE 2:**
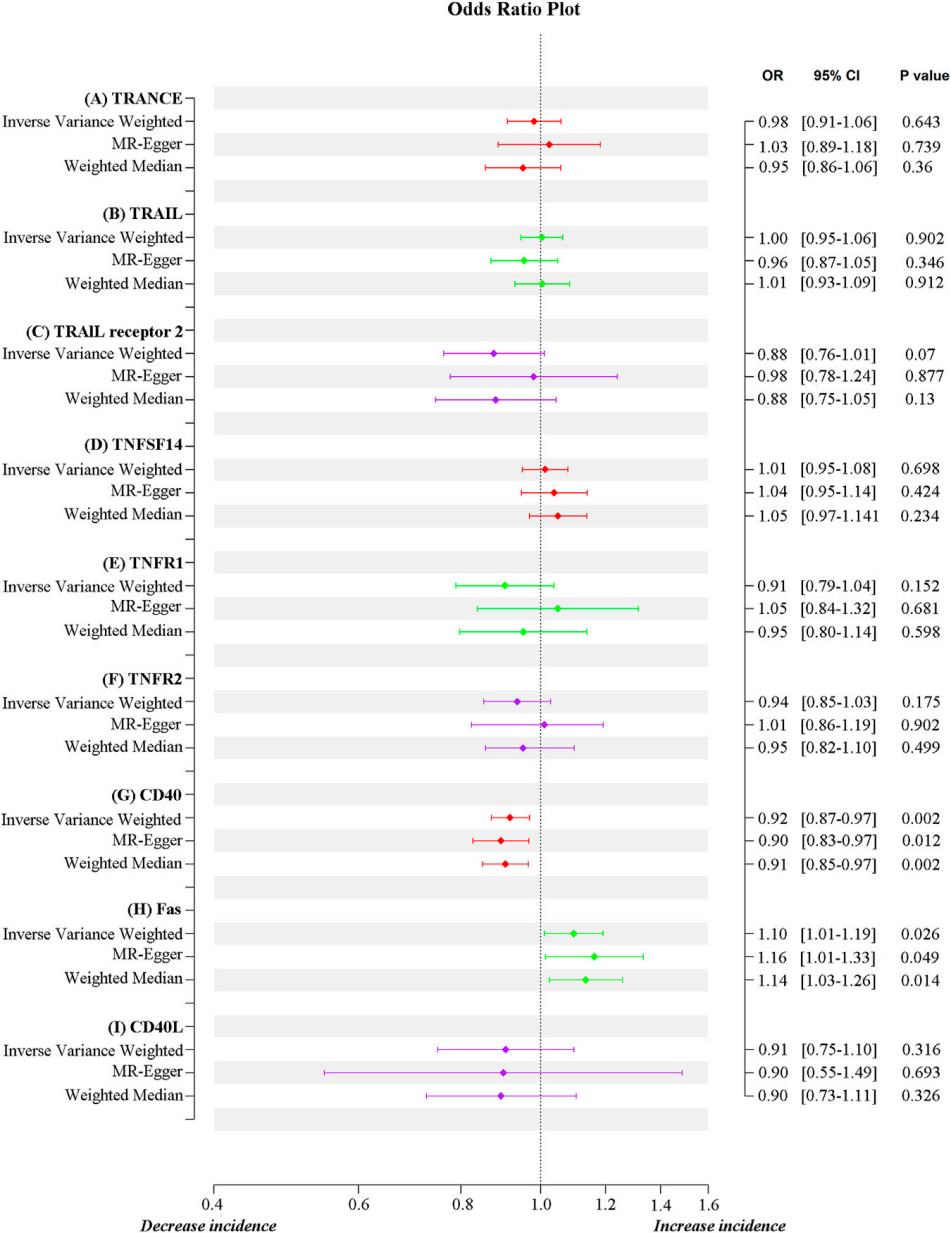
Correlation of TNF genetic predisposition with severe COVID-19.

**FIGURE 3:**
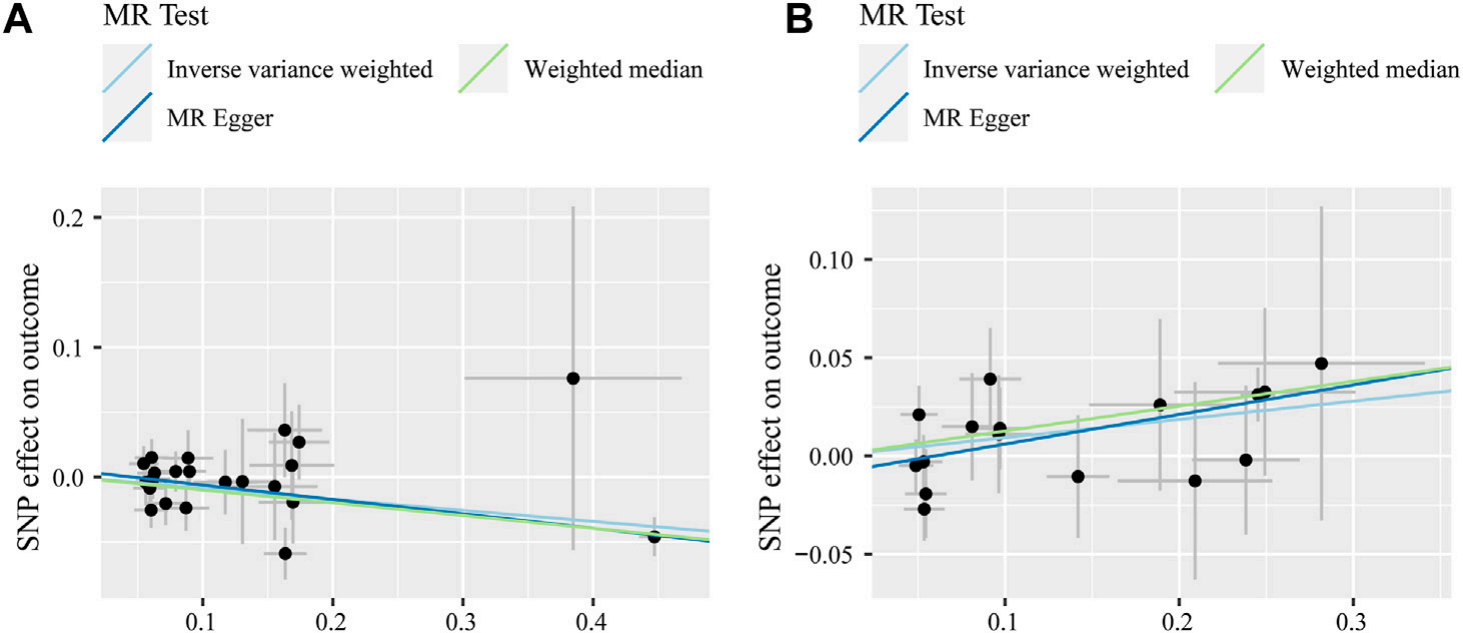
Scatter plot of SNPs associated with CD40 levels **(A)** and FAS levels **(B)** and severe COVID-19 after outlier removal with MR-PRESSO. Analyses were performed through inverse variance-weighted, weighted median, and MR‒Egger methods. The slope of each line corresponds to the estimated MR effect per method.

MR estimates for different methods to assess the causal impact of TNFs on severe COVID-19 are presented in Supplementary Table S3. As illustrated in Figure 2, genetically predicted CD40 levels were significantly negatively correlated with severe COVID-19 (IVW, OR = 0.92, 95% confidence interval (CI) = 0.87–0.97, *p* = 0.002). This result was also supported by MR‒Egger (OR = 0.90, 95% CI = 0.83–0.97, *p* = 0.012) and weighted median (OR = 0.91, 95% CI = 0.85–0.97, *p* = 0.002) analyses. Using the IVW approach under a multiplicative random-effects model, a positive correlation was detected between FAS (OR = 1.10, 95% CI = 1.01–1.19, *p* = 0.026) and confirmed very severe respiratory COVID-19, which was consistent with MR‒Egger (OR = 1.16, 95% CI = 1.01–1.33, *p* = 0.049) and weighted median (OR = 1.14, 95% CI = 1.03–1.26, *p* = 0.014) analyses. The estimated effect sizes of the SNPs on both exposures (CD40 and FAS) and outcomes (severe COVID-19) are presented in scatter plots in Figure 3.

Sensitivity analysis did not reveal heterogeneity (MR‒Egger, Q_P-val = 0.483; IVW, Q_P-val = 0.497) or significant horizontal pleiotropy (intercept = 0.005, se = 0.006, *p* = 0.388) for CD40 with severe COVID-19. Similarly, there was no clear evidence of heterogeneity (MR‒Egger, Q_P-val = 0.796; IVW, Q_P-val = 0.782) or horizontal pleiotropy (intercept = −0.009, se = 0.009, *p* = 0.322) between FAS and severe COVID-19 (Supplementary Table S4). Funnel plots, indicating directional horizontal pleiotropy for an outcome, are illustrated in Supplementary Figure S1, indicating that the results were robust and convincing. The results of the leave-one-out method showed that the causal relationship between CD40 and severe COVID-19 was robust, while FAS required caution (Supplementary Figure S2).

## 4 Discussion

Proteins circulating in the blood are critical for predicting human disease and are often used as biomarkers for clinical decision making (Folkersen et al., 2020). Previous studies have revealed the role of TNF superfamily members in cancer (Flieswasser et al., 2022; Curtis et al., 2023), autoimmune diseases (Tian et al., 2020; Chen et al., 2023), and respiratory diseases (Cho et al., 2020; Cross et al., 2023), which have been proven to be involved with severe COVID-19 (Zhang et al., 2021). Our genetic evidence suggests that FAS is positively associated with the severity of COVID-19 and that CD40 is protective against severe COVID-19. Given the global prevalence of COVID-19, this study may provide new evidence to explore biomarkers and drug targets for cases of severe disease.

The outcome of COVID-19 is thought to be associated with an excess of pro-inflammatory cytokines, leading to a “cytokine storm” and acute respiratory distress syndrome (Cron et al., 2023; Zebardast et al., 2023). FAS is a death receptor that mediates programmed apoptosis to maintain immune homeostasis. Supporting this hypothesis, patients with COVID-19 show elevated FAS expression on the T cell in plasma, which is known to be related to higher levels of caspase activation and PS exposure on the T cell and ultimately leads to T cell apoptosis, which is a feature of severe COVID-19 (Andre et al., 2022). Banerjee et al. (2022) showed that nedagliptin may significantly block TNF-induced inflammation/Fas-induced apoptotic death signaling compared to the positive control drug oseltamivir. This study may provide genetic evidence for the identification of COVID-19 biomarkers. Moreover, although the leave-one-out method showed that the causal relationship between FAS and severe cases of COVID-19 is not robust, this would not lead to a large bias in the results because FAS and severe COVID-19 are driven by potentially influential SNPs (F > 10).

The role of CD40 in COVID-19 is mainly applicable to vaccines. The mammalian innate immune response has a memory capacity called “trained immunity” (Netea et al., 2020). Murphy et al. (2023) used an adenoviral vector for the ChAdOx1 nCoV-19 vaccine (AZD1222) to observe whether it induces training immunity in humans and found that this vaccine induces metabolic reprogramming of monocytes and leads to enhanced expression of HLA-DR, CD40, and CD80 on monocytes, with many of the characteristics of trained immunity.Marlin et al. (2021) proposed a viral antigen vaccine targeting CD40-expressing antigen-presenting cells. They targeted the receptor-binding domain (RBD) of the SARS-CoV-2 spike protein toward CD40 (αCD40.RBD) to induce significant levels of specific T and B cells with a long-term memory phenotype. This vaccine significantly enhanced protection against a new high-dose toxicity challenge compared to unvaccinated recovery animals. These findings, combined with our results, exemplify the strong potential of CD40 in vaccine development. However, more work is needed to determine the role of CD40 in vaccine development for SARS-CoV-2.

This work adopted a solid quasi-experimental method grounded in international consortia and utilized large, high-quality GWAS data. The genetic instrument for TNFs consists of multiple SNPs that are robustly associated with each TNF, thus providing a powerful genetic tool. In addition, there are several limitations. First, the probability that TNF-associated SNPs impact severe COVID-19 outcomes via causal pathways apart from TNF levels cannot be completely excluded. Second, genetic variants related to severe COVID-19 were obtained from comparisons between severe COVID-19 and general cohorts. Information on the SARS-CoV-2 infection status of control participants was limited, which might influence the MR analyses in this study (Yao et al., 2022) (https://www.covid19hg.org/results/r7/). Third, the majority of the sample involved was European, which minimized the bias in population stratification. However, we must acknowledge that Europe is made up of very diverse ethnicities, and the applicability of the results obtained so far to different ethnicities needs further study. Nevertheless, as genetic variation is quite strongly related to exposure, the bias due to sample overlap is quite small.

## 5 Conclusion

In conclusion, this MR study provides genetic evidence supporting that increased expression of FAS may be associated with the risk of severe COVID-19, with a possible protective effect of CD40 against COVID-19.

## Data Availability

The datasets presented in this study can be found in online repositories. The names of the repository/repositories and accession number(s) can be found in the article/Supplementary Material.
