# Supplementary material for: Mendelian randomization analysis identified tumor necrosis factor as being associated with severe COVID-19
Source: Front Pharmacol. 2023 Jun 16;14:1171404. doi: 10.3389/fphar.2023.1171404 (PMC10311560; doi:10.3389/fphar.2023.1171404)
Supplement: Supplementary file 1 [file DataSheet1.docx]

Supplementary Material

Mendelian randomization analysis identified tumor necrosis factor associated with severe COVID-19

**Contents**

Table S1 Summary information on the genome-wide association studies used as data source.

Table S2 Instrument SNPs after harmonize for exposures and COVID-19 in the present study.

Table S3. Mendelian randomization estimates for the association of TNFs with severe COVID-19.

Table S4. Assessment of the presence of horizontal pleiotropy (MR-Egger intercept).

Table S5. Mendelian randomization estimates for the association of COVID-19 with exposures.

Figure S1. Funnel plot of SNPs associated with CD40 levels (A) and FAS levels (B) and severe COVID-19 after outliers removal with MR-PRESSO.

Figure S2. Plots of “leave-one-out” analyses for MR analyses of the causal effect of CD40 levels (A) and FAS levels (B) on severe COVID-19.

Table S1 Summary information on the genome-wide association studies used as data source.

| **Exposures** |  |  |  |
| --- | --- | --- | --- |
| **Phenotype** | **Description** | **Sample size** | **Population** |
| TRANCE | TNF-related activation-induced cytokine | 21758 | European |
| TRAIL | TNF-related apoptosis-inducing ligand | 21758 | European |
| TRAILR2 | TNF-related apoptosis-inducing ligand receptor 2 | 21758 | European |
| TNFSF14 | Tumor necrosis factor ligand superfamily member 14 | 21758 | European |
| TNFR1 | Tumor necrosis factor receptor 1 | 21758 | European |
| TNFR2 | Tumor necrosis factor receptor 2 | 21758 | European |
| CD40 | Tumor necrosis factor receptor superfamily member 5 | 21758 | European |
| FAS | Tumor necrosis factor receptor superfamily member 6 | 21758 | European |
| CD40L | CD40 ligand | 21758 | European |
| **Outcomes** |  |  |  |
| Severe COVID-19 | Very severe respiratory confirmed covid vs. population | 18,152/1,145,546 | European |

Table S2 Instrument SNPs after harmonize for exposures and COVID-19 in the present study.

| **TNF-related activation-induced cytokine levels and severe covid-19** | | | | | | | | | | | | | | | |
| --- | --- | --- | --- | --- | --- | --- | --- | --- | --- | --- | --- | --- | --- | --- | --- |
|  | **Exposure** | | | | | | | **Outcome** | | | | | |  | |
| **SNP** | **Effect allele** | **Other allele** | **beta** | **se** | ***Eaf*** | ***P*-val** | **Effect allele** | | **Other allele** | **beta** | **se** | ***P*-val** | **R²** | | **F-statistic** |
| rs10746949 | T | C | 0.0749 | 0.0161 | 0.8052 | 3.36E-06 | T | | C | 0.0189 | 0.0166 | 0.2548 | 0.0018 | | 38.3557 |
| rs11065979 | T | C | 0.0565 | 0.0109 | 0.4257 | 2.26E-07 | T | | C | -0.0222 | 0.0134 | 0.0972 | 0.0016 | | 34.0116 |
| rs117113213 | A | G | -0.1743 | 0.0292 | 0.0484 | 2.38E-09 | A | | G | 0.0278 | 0.0497 | 0.5752 | 0.0028 | | 61.0549 |
| rs11713634 | A | G | -0.105 | 0.016 | 0.2882 | 5.55E-11 | A | | G | 0.0051 | 0.0146 | 0.7250 | 0.0045 | | 98.8573 |
| rs1577570 | C | T | 0.1444 | 0.0302 | 0.9538 | 1.75E-06 | C | | T | -0.0055 | 0.0287 | 0.8468 | 0.0018 | | 40.0536 |
| rs17062089 | T | C | -0.2366 | 0.0517 | 0.0182 | 4.74E-06 | T | | C | -0.0400 | 0.0552 | 0.4687 | 0.0020 | | 43.6117 |
| rs17864557 | T | G | -0.071 | 0.0152 | 0.1649 | 3.24E-06 | T | | G | -0.0089 | 0.0171 | 0.6052 | 0.0014 | | 30.2474 |
| rs2071352 | A | T | 0.0953 | 0.0182 | 0.1369 | 1.55E-07 | A | | T | 0.0535 | 0.0170 | 0.0016 | 0.0021 | | 46.7943 |
| rs2242313 | G | C | -0.1669 | 0.0111 | 0.441 | 9.19E-51 | G | | C | -0.0109 | 0.0135 | 0.4214 | 0.0137 | | 302.9549 |
| rs4512994 | C | A | -0.1208 | 0.0108 | 0.5377 | 4.62E-29 | C | | A | 0.0036 | 0.0126 | 0.7726 | 0.0073 | | 158.9897 |
| rs4563544 | T | G | 0.1265 | 0.0266 | 0.9071 | 1.98E-06 | T | | G | 0.0177 | 0.0332 | 0.5946 | 0.0027 | | 58.8347 |
| rs516649 | C | T | -0.0627 | 0.0121 | 0.7121 | 2.04E-07 | C | | T | 0.0261 | 0.0148 | 0.0773 | 0.0016 | | 35.1259 |
| rs704 | A | G | -0.1073 | 0.011 | 0.4753 | 1.39E-22 | A | | G | 0.0061 | 0.0125 | 0.6231 | 0.0057 | | 125.6575 |
| rs7279433 | G | A | 0.0595 | 0.0129 | 0.6659 | 4.25E-06 | G | | A | -0.0088 | 0.0203 | 0.6665 | 0.0016 | | 34.3252 |
| rs73224404 | G | A | -0.1416 | 0.015 | 0.2082 | 2.82E-21 | G | | A | -0.0264 | 0.0173 | 0.1271 | 0.0066 | | 144.7813 |
| rs76350197 | C | G | -0.133 | 0.0287 | 0.0499 | 3.68E-06 | C | | G | 0.0361 | 0.0378 | 0.3400 | 0.0017 | | 36.5520 |
| rs78940328 | G | A | -0.1333 | 0.028 | 0.0545 | 2.00E-06 | G | | A | -0.0188 | 0.0280 | 0.5017 | 0.0018 | | 39.9138 |
| rs79287178 | A | G | -0.5487 | 0.0348 | 0.0332 | 3.91922E-56 | A | | G | 0.0293 | 0.0418 | 0.4833 | 0.0193 | | 428.7752 |
| rs9266638 | T | C | -0.0585 | 0.0121 | 0.2999 | 1.36101E-06 | T | | C | 0.0064 | 0.0176 | 0.7168 | 0.0014 | | 31.3099 |
| rs9805907 | T | C | -0.0612 | 0.0133 | 0.3271 | 0.000004517 | T | | C | 0.0116 | 0.0141 | 0.4117 | 0.0016 | | 35.9302 |
| rs987814 | A | C | 0.0498 | 0.0108 | 0.5157 | 4.17398E-06 | A | | C | 0.0065 | 0.0126 | 0.6096 | 0.0012 | | 26.9847 |
|  |  |  |  |  |  |  |  | |  |  |  |  |  | |  |
| **TNF-related apoptosis-inducing ligand levels and severe covid-19** | | | | | | | | | | | | | | | |
|  | **Exposure** | | | | | | | **Outcome** | | | | | |  | |
| **SNP** | **Effect allele** | **Other allele** | **beta** | **se** | ***Eaf*** | ***P*-val** | **Effect allele** | | **Other allele** | **beta** | **se** | ***P*-val** | **R²** | | **F-statistic** |
| rs10502 | C | G | 0.0574 | 0.0117 | 0.3911 | 9.57503E-07 | C | | G | 0.0028 | 0.0133 | 0.8333 | 0.0016 | | 34.1939 |
| rs1108552 | G | T | -0.0621 | 0.0135 | 0.28 | 4.01098E-06 | G | | T | -0.0036 | 0.0151 | 0.8120 | 0.0016 | | 33.8812 |
| rs112311125 | T | C | -0.1859 | 0.0396 | 0.0258 | 2.74897E-06 | T | | C | 0.0315 | 0.0432 | 0.4651 | 0.0017 | | 37.8609 |
| rs115358588 | G | T | -0.1825 | 0.0385 | 0.0323 | 2.10499E-06 | G | | T | 0.0554 | 0.0362 | 0.1255 | 0.0021 | | 45.3924 |
| rs11648622 | A | G | 0.0688 | 0.0127 | 0.2701 | 6.03893E-08 | A | | G | 0.0139 | 0.0149 | 0.3512 | 0.0019 | | 40.6804 |
| rs116701166 | A | G | -0.1458 | 0.0311 | 0.0585 | 0.000002719 | A | | G | -0.0992 | 0.0428 | 0.0206 | 0.0023 | | 51.0644 |
| rs117538711 | G | A | -0.1663 | 0.0361 | 0.0428 | 4.18803E-06 | G | | A | 0.0029 | 0.0321 | 0.9272 | 0.0023 | | 49.4112 |
| rs142552223 | A | G | -0.4708 | 0.0324 | 0.0359 | 6.71738E-48 | A | | G | -0.0510 | 0.0479 | 0.2861 | 0.0153 | | 339.0109 |
| rs17265788 | C | G | -0.0736 | 0.0158 | 0.2091 | 3.09799E-06 | C | | G | 0.0327 | 0.0161 | 0.0427 | 0.0018 | | 39.0498 |
| rs17887098 | T | C | 0.2314 | 0.0503 | 0.021 | 0.000004138 | T | | C | -0.0970 | 0.0569 | 0.0883 | 0.0022 | | 48.0059 |
| rs199818304 | A | T | -0.0731 | 0.0155 | 0.5363 | 2.23599E-06 | A | | T | -0.0052 | 0.0131 | 0.6898 | 0.0027 | | 57.9755 |
| rs2304456 | G | T | -0.2581 | 0.0188 | 0.1143 | 5.14044E-43 | G | | T | 0.0107 | 0.0200 | 0.5914 | 0.0135 | | 297.4510 |
| rs232001 | G | C | -0.0902 | 0.0132 | 0.6345 | 8.1358E-12 | G | | C | -0.0086 | 0.0140 | 0.5395 | 0.0038 | | 82.4106 |
| rs2727271 | T | A | 0.0818 | 0.0158 | 0.1651 | 2.37498E-07 | T | | A | 0.0151 | 0.0199 | 0.4491 | 0.0018 | | 40.2068 |
| rs28929474 | T | C | 0.6551 | 0.0433 | 0.0202 | 1.18713E-51 | T | | C | -0.0223 | 0.0582 | 0.7012 | 0.0170 | | 375.9708 |
| rs34225068 | C | T | -0.1136 | 0.0236 | 0.8778 | 1.45499E-06 | C | | T | 0.0270 | 0.0204 | 0.1864 | 0.0028 | | 60.3999 |
| rs35617250 | T | C | 0.1125 | 0.0148 | 0.1826 | 2.62603E-14 | T | | C | -0.0079 | 0.0157 | 0.6147 | 0.0038 | | 82.5075 |
| rs4429452 | T | C | -0.087 | 0.0173 | 0.1266 | 4.93901E-07 | T | | C | -0.0100 | 0.0205 | 0.6248 | 0.0017 | | 36.4772 |
| rs4760 | G | A | -0.363 | 0.017 | 0.1608 | 1.6596E-101 | G | | A | -0.0105 | 0.0196 | 0.5938 | 0.0356 | | 802.2317 |
| rs546010680 | C | A | -0.1693 | 0.0356 | 0.0633 | 1.94402E-06 | C | | A | 0.0422 | 0.0387 | 0.2760 | 0.0034 | | 74.2003 |
| rs57250383 | T | C | -0.0596 | 0.0128 | 0.2866 | 3.10499E-06 | T | | C | -0.0250 | 0.0149 | 0.0936 | 0.0015 | | 31.6477 |
| rs673408 | G | A | -0.0865 | 0.0117 | 0.462 | 1.51217E-13 | G | | A | -0.0123 | 0.0126 | 0.3293 | 0.0037 | | 81.2239 |
| rs6843171 | A | G | -0.067 | 0.0133 | 0.3903 | 5.14802E-07 | A | | G | -0.0062 | 0.0138 | 0.6558 | 0.0021 | | 46.5803 |
| rs6888399 | C | G | -0.0608 | 0.0128 | 0.7204 | 2.18801E-06 | C | | G | -0.0114 | 0.0154 | 0.4592 | 0.0015 | | 32.4470 |
| rs6988771 | G | A | -0.0561 | 0.0121 | 0.4042 | 0.000003571 | G | | A | -0.0165 | 0.0134 | 0.2201 | 0.0015 | | 33.0286 |
| rs73563321 | T | C | -0.0893 | 0.0192 | 0.1061 | 3.24302E-06 | T | | C | 0.0136 | 0.0211 | 0.5203 | 0.0015 | | 32.9590 |
| rs79421282 | A | C | 0.0833 | 0.0181 | 0.158 | 4.21803E-06 | A | | C | -0.0019 | 0.0251 | 0.9404 | 0.0018 | | 40.2412 |
| rs7995392 | T | A | -0.1641 | 0.0353 | 0.0359 | 3.39297E-06 | T | | A | 0.0581 | 0.0341 | 0.0884 | 0.0019 | | 40.6306 |
| rs8178824 | T | C | 0.2642 | 0.0352 | 0.0302 | 5.84252E-14 | T | | C | -0.0279 | 0.0450 | 0.5354 | 0.0041 | | 89.3189 |
| rs9893227 | A | G | 0.1148 | 0.0247 | 0.0984 | 3.26498E-06 | A | | G | -0.0001 | 0.0190 | 0.9973 | 0.0023 | | 50.9939 |
|  |  |  |  |  |  |  |  | |  |  |  |  |  | |  |
| **TNF-related apoptosis-inducing ligand receptor 2 and severe covid-19** | | | | | | | | | | | | | | | |
|  | **Exposure** | | | | | | | **Outcome** | | | | | |  | |
| **SNP** | **Effect allele** | **Other allele** | **beta** | **se** | ***Eaf*** | ***P*-val** | **Effect allele** | | **Other allele** | **beta** | **se** | ***P*-val** | **R²** | | **F-statistic** |
| rs111634572 | T | C | -0.2792 | 0.0593 | 0.0156 | 0.0000025 | T | | C | 0.0620 | 0.0569 | 0.2758 | 0.0024 | | 52.2128 |
| rs117131030 | T | C | -0.2055 | 0.0428 | 0.0227 | 1.61002E-06 | T | | C | 0.0255 | 0.0489 | 0.6025 | 0.0019 | | 40.8414 |
| rs150209488 | T | A | 0.2309 | 0.0468 | 0.0181 | 8.19502E-07 | T | | A | 0.0693 | 0.0933 | 0.4581 | 0.0019 | | 41.3073 |
| rs2270108 | C | A | 0.0498 | 0.0106 | 0.3433 | 0.000002474 | C | | A | -0.0572 | 0.0140 | 0.0000 | 0.0011 | | 24.3554 |
| rs2293400 | T | A | 0.3074 | 0.0117 | 0.3376 | 3.4277E-152 | T | | A | 0.0060 | 0.0145 | 0.6816 | 0.0423 | | 960.0487 |
| rs2430796 | C | G | 0.1798 | 0.0223 | 0.9272 | 7.29625E-16 | C | | G | 0.0081 | 0.0239 | 0.7355 | 0.0044 | | 95.3658 |
| rs2430949 | A | G | 0.071 | 0.0154 | 0.1547 | 3.89404E-06 | A | | G | -0.0185 | 0.0180 | 0.3039 | 0.0013 | | 28.7210 |
| rs2506732 | T | C | 0.1236 | 0.0253 | 0.9446 | 1.07599E-06 | T | | C | -0.0168 | 0.0311 | 0.5888 | 0.0016 | | 34.8416 |
| rs35340437 | A | T | 0.0702 | 0.0151 | 0.1371 | 3.31299E-06 | A | | T | 0.0225 | 0.0194 | 0.2451 | 0.0012 | | 25.3973 |
| rs41308108 | A | G | -0.4582 | 0.0597 | 0.0119 | 1.69083E-14 | A | | G | 0.0578 | 0.0500 | 0.2473 | 0.0049 | | 107.9485 |
| rs4871846 | G | C | -0.071 | 0.0114 | 0.3987 | 5.53694E-10 | G | | C | 0.0205 | 0.0141 | 0.1438 | 0.0024 | | 52.7126 |
| rs58244673 | T | G | -0.0856 | 0.018 | 0.1078 | 1.98002E-06 | T | | G | -0.0129 | 0.0193 | 0.5022 | 0.0014 | | 30.7079 |
| rs62406826 | G | A | 0.0974 | 0.0211 | 0.0649 | 0.00000389 | G | | A | -0.0060 | 0.0274 | 0.8268 | 0.0012 | | 25.0801 |
| rs701455 | A | G | 0.0506 | 0.0107 | 0.5362 | 0.000002186 | A | | G | -0.0039 | 0.0128 | 0.7586 | 0.0013 | | 27.7409 |
| rs72680238 | A | C | 0.2151 | 0.0471 | 0.0215 | 4.84496E-06 | A | | C | -0.0896 | 0.1084 | 0.4081 | 0.0019 | | 42.4361 |
| rs79287178 | A | G | 0.1768 | 0.0322 | 0.0337 | 3.88803E-08 | A | | G | 0.0293 | 0.0418 | 0.4833 | 0.0020 | | 44.3813 |
| rs7946636 | A | G | 0.0521 | 0.0114 | 0.6668 | 4.85803E-06 | A | | G | -0.0132 | 0.0136 | 0.3331 | 0.0012 | | 26.2730 |
| rs9355236 | C | A | -0.1167 | 0.0255 | 0.0608 | 4.83604E-06 | C | | A | -0.0244 | 0.0267 | 0.3600 | 0.0016 | | 33.8913 |
| rs9845717 | T | C | 0.0637 | 0.0138 | 0.7779 | 0.000004028 | T | | C | -0.0066 | 0.0163 | 0.6853 | 0.0014 | | 30.5471 |
|  |  |  |  |  |  |  |  | |  |  |  |  |  | |  |
| **Tumor necrosis factor ligand superfamily member 14 and severe covid-19** | | | | | | | | | | | | | | | |
|  | **Exposure** | | | | | | | **Outcome** | | | | | |  | |
| **SNP** | **Effect allele** | **Other allele** | **beta** | **se** | ***Eaf*** | ***P*-val** | **Effect allele** | | **Other allele** | **beta** | **se** | ***P*-val** | **R²** | | **F-statistic** |
| rs10016980 | A | T | 0.0591 | 0.0127 | 0.4687 | 0.000003176 | A | | T | 0.0057 | 0.0126 | 0.6528 | 0.0017 | | 37.9118 |
| rs11085200 | T | G | 0.0714 | 0.0141 | 0.3068 | 4.16303E-07 | T | | G | -0.0071 | 0.0227 | 0.7533 | 0.0022 | | 47.2783 |
| rs11738703 | T | A | -0.0622 | 0.0129 | 0.255 | 0.000001309 | T | | A | -0.0019 | 0.0144 | 0.8966 | 0.0015 | | 32.0277 |
| rs138416186 | T | G | -0.2388 | 0.0523 | 0.0174 | 4.97096E-06 | T | | G | 0.0616 | 0.0527 | 0.2429 | 0.0019 | | 42.5061 |
| rs138811446 | T | C | 0.2163 | 0.0301 | 0.0517 | 6.60693E-13 | T | | C | -0.0172 | 0.0315 | 0.5850 | 0.0046 | | 100.2664 |
| rs140343644 | A | G | 0.187 | 0.0269 | 0.0484 | 3.8089E-12 | A | | G | -0.0563 | 0.0318 | 0.0763 | 0.0032 | | 70.3061 |
| rs148392103 | A | G | 0.3333 | 0.0729 | 0.0099 | 4.87405E-06 | A | | G | -0.0502 | 0.0692 | 0.4684 | 0.0022 | | 47.4833 |
| rs1540198 | G | C | -0.0749 | 0.0162 | 0.8147 | 3.95804E-06 | G | | C | 0.0188 | 0.0299 | 0.5291 | 0.0017 | | 36.9132 |
| rs16886825 | G | A | 0.1107 | 0.024 | 0.0696 | 3.82296E-06 | G | | A | -0.0142 | 0.0280 | 0.6116 | 0.0016 | | 34.5838 |
| rs2473851 | G | A | -0.0619 | 0.0121 | 0.4402 | 3.27703E-07 | G | | A | -0.0080 | 0.0179 | 0.6542 | 0.0019 | | 41.1618 |
| rs2548874 | A | G | -0.0551 | 0.0119 | 0.5584 | 3.44802E-06 | A | | G | 0.0143 | 0.0127 | 0.2629 | 0.0015 | | 32.6240 |
| rs2986744 | A | G | 0.1206 | 0.0255 | 0.0741 | 2.16098E-06 | A | | G | 0.0095 | 0.0244 | 0.6960 | 0.0020 | | 43.5064 |
| rs344560 | C | T | 0.6301 | 0.0252 | 0.9397 | 1.5886E-137 | C | | T | 0.0386 | 0.0273 | 0.1581 | 0.0450 | | 1025.0109 |
| rs344576 | T | C | 0.1389 | 0.0192 | 0.1185 | 4.90795E-13 | T | | C | 0.0037 | 0.0213 | 0.8634 | 0.0040 | | 88.0457 |
| rs4809565 | C | T | 0.0644 | 0.0139 | 0.7 | 3.71501E-06 | C | | T | 0.0144 | 0.0143 | 0.3137 | 0.0017 | | 37.9627 |
| rs58645166 | A | G | -0.0839 | 0.0182 | 0.1669 | 0.000004157 | A | | G | -0.0004 | 0.0230 | 0.9862 | 0.0020 | | 42.6715 |
| rs6118670 | G | A | -0.1439 | 0.031 | 0.9555 | 0.000003378 | G | | A | 0.0366 | 0.0356 | 0.3047 | 0.0018 | | 38.3784 |
| rs6945477 | C | T | -0.0938 | 0.0186 | 0.1084 | 4.90501E-07 | C | | T | -0.0093 | 0.0203 | 0.6461 | 0.0017 | | 37.0641 |
| rs763419 | G | C | -0.0772 | 0.0162 | 0.1665 | 2.04498E-06 | G | | C | -0.0113 | 0.0181 | 0.5333 | 0.0017 | | 36.0481 |
| rs77509297 | T | C | 0.2122 | 0.0459 | 0.0217 | 3.69496E-06 | T | | C | -0.0044 | 0.0519 | 0.9330 | 0.0019 | | 41.6738 |
| rs78565612 | T | C | -0.2233 | 0.0489 | 0.0249 | 4.91598E-06 | T | | C | -0.0254 | 0.0477 | 0.5942 | 0.0024 | | 52.8066 |
| rs8046862 | G | A | -0.1702 | 0.0355 | 0.0591 | 0.000001585 | G | | A | -0.0030 | 0.0255 | 0.9068 | 0.0032 | | 70.3170 |
|  |  |  |  |  |  |  |  | |  |  |  |  |  | |  |
| **Tumor necrosis factor receptor 1 and severe covid-19** | | | | | | | | | | | | | | | |
|  | **Exposure** | | | | | | | **Outcome** | | | | | |  | |
| **SNP** | **Effect allele** | **Other allele** | **beta** | **se** | ***Eaf*** | ***P*-val** | **Effect allele** | | **Other allele** | **beta** | **se** | ***P*-val** | **R²** | | **F-statistic** |
| rs10255400 | A | G | -0.0468 | 0.0099 | 0.6164 | 2.34401E-06 | A | | G | 0.0139 | 0.0178 | 0.4353 | 0.0010 | | 22.5576 |
| rs111693226 | T | C | -0.2874 | 0.0519 | 0.0216 | 3.00898E-08 | T | | C | -0.0326 | 0.0428 | 0.4459 | 0.0035 | | 76.2205 |
| rs1130094 | C | T | 0.0638 | 0.0097 | 0.4889 | 5.63378E-11 | C | | T | -0.0340 | 0.0128 | 0.0080 | 0.0020 | | 44.3466 |
| rs113602289 | T | G | -0.2697 | 0.0576 | 0.0159 | 2.86299E-06 | T | | G | -0.0629 | 0.1324 | 0.6348 | 0.0023 | | 49.6360 |
| rs114659025 | T | C | -0.0837 | 0.0171 | 0.0707 | 9.60196E-07 | T | | C | -0.0151 | 0.0252 | 0.5485 | 0.0009 | | 20.0463 |
| rs117315715 | A | G | -0.2623 | 0.0566 | 0.0148 | 3.54299E-06 | A | | G | 0.0125 | 0.1465 | 0.9318 | 0.0020 | | 43.7385 |
| rs13329966 | G | T | -0.0689 | 0.0149 | 0.1039 | 3.79001E-06 | G | | T | -0.0346 | 0.0211 | 0.1017 | 0.0009 | | 19.2488 |
| rs13338374 | C | G | -0.0641 | 0.0127 | 0.1595 | 4.55995E-07 | C | | G | -0.0196 | 0.0189 | 0.3003 | 0.0011 | | 23.9940 |
| rs16917106 | A | C | 0.0515 | 0.0109 | 0.2306 | 2.27499E-06 | A | | C | -0.0076 | 0.0158 | 0.6318 | 0.0009 | | 20.4948 |
| rs17054989 | A | G | -0.0817 | 0.0178 | 0.0567 | 4.66402E-06 | A | | G | 0.0253 | 0.0247 | 0.3059 | 0.0007 | | 15.5452 |
| rs182318272 | A | G | 0.109 | 0.0238 | 0.0576 | 4.59801E-06 | A | | G | -0.0597 | 0.0301 | 0.0472 | 0.0013 | | 28.0983 |
| rs200417836 | C | T | -0.2744 | 0.0595 | 0.0285 | 3.95103E-06 | C | | T | -0.0039 | 0.0408 | 0.9245 | 0.0042 | | 91.0918 |
| rs2093916 | C | T | 0.0864 | 0.0167 | 0.9132 | 2.48199E-07 | C | | T | -0.0250 | 0.0205 | 0.2211 | 0.0012 | | 25.7772 |
| rs2530657 | C | G | -0.1329 | 0.0288 | 0.9638 | 3.91598E-06 | C | | G | 0.0044 | 0.0598 | 0.9413 | 0.0012 | | 26.8466 |
| rs4149584 | T | C | -0.3503 | 0.0384 | 0.0192 | 7.92866E-20 | T | | C | 0.0247 | 0.0550 | 0.6528 | 0.0046 | | 101.0143 |
| rs4976646 | C | T | 0.0482 | 0.0099 | 0.3467 | 1.18201E-06 | C | | T | 0.0014 | 0.0133 | 0.9142 | 0.0011 | | 22.9206 |
| rs7611124 | C | T | -0.0576 | 0.0116 | 0.1907 | 6.62995E-07 | C | | T | 0.0270 | 0.0162 | 0.0952 | 0.0010 | | 22.3028 |
| rs76620115 | A | T | -0.2622 | 0.0511 | 0.0165 | 2.87098E-07 | A | | T | 0.0828 | 0.1012 | 0.4131 | 0.0022 | | 48.6522 |
|  |  |  |  |  |  |  |  | |  |  |  |  |  | |  |
| **Tumor necrosis factor receptor 2 and severe covid-19** | | | | | | | | | | | | | | | |
|  | **Exposure** | | | | | | | **Outcome** | | | | | |  | |
| **SNP** | **Effect allele** | **Other allele** | **beta** | **se** | ***Eaf*** | ***P*-val** | **Effect allele** | | **Other allele** | **beta** | **se** | ***P*-val** | **R²** | | **F-statistic** |
| rs1078397 | C | G | -0.0554 | 0.0106 | 0.3419 | 1.65699E-07 | C | | G | -0.0043 | 0.0142 | 0.7616 | 0.0014 | | 30.0898 |
| rs11066320 | G | A | -0.0524 | 0.0094 | 0.5786 | 2.89101E-08 | G | | A | 0.0215 | 0.0135 | 0.1111 | 0.0013 | | 29.1693 |
| rs111693226 | T | C | -0.3038 | 0.0519 | 0.0215 | 4.84094E-09 | T | | C | -0.0326 | 0.0428 | 0.4459 | 0.0039 | | 84.8152 |
| rs112758380 | A | G | 0.0992 | 0.0213 | 0.0617 | 3.10399E-06 | A | | G | -0.0607 | 0.0460 | 0.1875 | 0.0011 | | 24.8173 |
| rs113602289 | T | G | -0.2651 | 0.0577 | 0.0158 | 4.39097E-06 | T | | G | -0.0629 | 0.1324 | 0.6348 | 0.0022 | | 47.6562 |
| rs114508013 | A | G | -0.1387 | 0.03 | 0.0305 | 3.88401E-06 | A | | G | -0.0244 | 0.0457 | 0.5929 | 0.0011 | | 24.7802 |
| rs1201165 | G | T | -0.0593 | 0.0106 | 0.5807 | 1.911E-08 | G | | T | 0.0238 | 0.0140 | 0.0896 | 0.0017 | | 37.3198 |
| rs12542796 | T | C | -0.052 | 0.0111 | 0.6138 | 2.82599E-06 | T | | C | -0.0214 | 0.0143 | 0.1364 | 0.0013 | | 27.9262 |
| rs12669946 | G | A | 0.0561 | 0.0112 | 0.4448 | 5.91902E-07 | G | | A | 0.0048 | 0.0137 | 0.7241 | 0.0016 | | 33.8707 |
| rs13149164 | A | G | -0.0935 | 0.0204 | 0.0508 | 4.68803E-06 | A | | G | -0.0739 | 0.0483 | 0.1261 | 0.0008 | | 18.3578 |
| rs13164856 | C | T | -0.0488 | 0.0105 | 0.2945 | 3.18999E-06 | C | | T | 0.0011 | 0.0148 | 0.9402 | 0.0010 | | 21.5507 |
| rs140330160 | T | C | -0.2287 | 0.0494 | 0.0145 | 3.61801E-06 | T | | C | 0.0581 | 0.0742 | 0.4339 | 0.0015 | | 32.5698 |
| rs150434093 | A | G | -0.2874 | 0.0587 | 0.0171 | 9.9481E-07 | A | | G | -0.1087 | 0.1037 | 0.2946 | 0.0028 | | 60.5753 |
| rs17437248 | A | G | -0.06 | 0.0129 | 0.1427 | 3.38501E-06 | A | | G | -0.0015 | 0.0215 | 0.9450 | 0.0009 | | 19.1801 |
| rs1867561 | C | G | -0.0611 | 0.0133 | 0.777 | 4.33701E-06 | C | | G | 0.0129 | 0.0227 | 0.5687 | 0.0013 | | 28.1825 |
| rs187028 | T | A | -0.0484 | 0.0104 | 0.6927 | 3.39203E-06 | T | | A | -0.0247 | 0.0142 | 0.0821 | 0.0010 | | 21.7191 |
| rs2093916 | C | T | 0.0822 | 0.0168 | 0.9133 | 1.04901E-06 | C | | T | -0.0250 | 0.0205 | 0.2211 | 0.0011 | | 23.3051 |
| rs2256974 | A | C | 0.0729 | 0.0119 | 0.1825 | 9.49795E-10 | A | | C | -0.0266 | 0.0163 | 0.1038 | 0.0016 | | 34.5544 |
| rs28399563 | A | G | 0.0657 | 0.0116 | 0.1949 | 1.36101E-08 | A | | G | -0.0117 | 0.0235 | 0.6173 | 0.0014 | | 29.5114 |
| rs4330937 | G | T | -0.0505 | 0.0109 | 0.7181 | 3.48001E-06 | G | | T | -0.0091 | 0.0152 | 0.5505 | 0.0010 | | 22.4864 |
| rs4647865 | A | G | -0.0592 | 0.0112 | 0.4931 | 1.239E-07 | A | | G | 0.0125 | 0.0196 | 0.5247 | 0.0018 | | 38.1831 |
| rs4664326 | C | T | -0.0439 | 0.0095 | 0.5633 | 3.76999E-06 | C | | T | 0.0160 | 0.0127 | 0.2061 | 0.0009 | | 20.6478 |
| rs4763305 | T | C | -0.0479 | 0.0104 | 0.6093 | 4.20301E-06 | T | | C | -0.0042 | 0.0138 | 0.7620 | 0.0011 | | 23.7919 |
| rs4766578 | A | T | -0.0736 | 0.0101 | 0.5246 | 3.83884E-13 | A | | T | 0.0230 | 0.0134 | 0.0867 | 0.0027 | | 58.9423 |
| rs4897099 | A | G | -0.0456 | 0.0095 | 0.426 | 0.000001563 | A | | G | 0.0264 | 0.0134 | 0.0488 | 0.0010 | | 22.1463 |
| rs4976646 | C | T | 0.0564 | 0.01 | 0.3464 | 1.57601E-08 | C | | T | 0.0014 | 0.0133 | 0.9142 | 0.0014 | | 31.3822 |
| rs5746026 | A | G | -0.3758 | 0.0281 | 0.0365 | 1.06512E-40 | A | | G | 0.0335 | 0.0370 | 0.3662 | 0.0099 | | 218.2743 |
| rs61747728 | T | C | 0.18 | 0.0297 | 0.0396 | 1.33199E-09 | T | | C | 0.0247 | 0.0368 | 0.5017 | 0.0025 | | 53.7493 |
| rs72993079 | T | C | 0.118 | 0.025 | 0.0638 | 0.000002329 | T | | C | -0.0229 | 0.0292 | 0.4317 | 0.0017 | | 36.2481 |
| rs949686 | C | G | -0.0843 | 0.0184 | 0.0576 | 4.65404E-06 | C | | G | -0.0554 | 0.0261 | 0.0342 | 0.0008 | | 16.7980 |
| rs9897524 | T | C | -0.0549 | 0.0115 | 0.3267 | 1.72898E-06 | T | | C | -0.0049 | 0.0149 | 0.7412 | 0.0013 | | 28.8860 |
|  |  |  |  |  |  |  |  | |  |  |  |  |  | |  |
| **Tumor necrosis factor receptor superfamily member 5 and severe covid-19** | | | | | | | | | | | | | | | |
|  | **Exposure** | | | | | | | **Outcome** | | | | | |  | |
| **SNP** | **Effect allele** | **Other allele** | **beta** | **se** | ***Eaf*** | ***P*-val** | **Effect allele** | | **Other allele** | **beta** | **se** | ***P*-val** | **R²** | | **F-statistic** |
| rs10218647 | A | G | 0.0607 | 0.013 | 0.7181 | 3.03201E-06 | A | | G | 0.0150 | 0.0143 | 0.2952 | 0.0015 | | 32.5023 |
| rs11211138 | G | A | -0.0562 | 0.0119 | 0.6698 | 0.000002123 | G | | A | 0.0041 | 0.0178 | 0.8180 | 0.0014 | | 30.4376 |
| rs116198125 | C | T | 0.1553 | 0.0329 | 0.042 | 2.36499E-06 | C | | T | -0.0071 | 0.0414 | 0.8631 | 0.0019 | | 42.3068 |
| rs12610383 | A | G | -0.1305 | 0.0269 | 0.0743 | 1.20701E-06 | A | | G | 0.0035 | 0.0482 | 0.9425 | 0.0023 | | 51.0867 |
| rs13064530 | A | G | 0.0629 | 0.0133 | 0.235 | 2.16601E-06 | A | | G | 0.0032 | 0.0146 | 0.8282 | 0.0014 | | 30.9926 |
| rs1321001 | G | T | -0.0715 | 0.0154 | 0.1726 | 3.22998E-06 | G | | T | 0.0203 | 0.0167 | 0.2253 | 0.0015 | | 31.8135 |
| rs2120295 | G | A | -0.0871 | 0.0179 | 0.1686 | 1.22301E-06 | G | | A | 0.0237 | 0.0176 | 0.1773 | 0.0021 | | 46.3701 |
| rs2239534 | A | C | 0.0792 | 0.0141 | 0.1978 | 1.77101E-08 | A | | C | 0.0044 | 0.0153 | 0.7758 | 0.0020 | | 43.3944 |
| rs2561033 | C | T | -0.0616 | 0.0132 | 0.2474 | 2.94503E-06 | C | | T | 0.0014 | 0.0154 | 0.9271 | 0.0014 | | 30.7857 |
| rs2963651 | T | G | -0.0603 | 0.013 | 0.4353 | 3.31101E-06 | T | | G | 0.0254 | 0.0136 | 0.0625 | 0.0018 | | 38.9607 |
| rs404842 | G | A | 0.0593 | 0.0127 | 0.3604 | 3.04698E-06 | G | | A | -0.0085 | 0.0141 | 0.5488 | 0.0016 | | 35.3278 |
| rs45456397 | A | C | -0.1173 | 0.0247 | 0.074 | 2.12501E-06 | A | | C | 0.0039 | 0.0249 | 0.8761 | 0.0019 | | 41.1024 |
| rs4801216 | T | C | -0.0898 | 0.0127 | 0.6029 | 1.76604E-12 | T | | C | -0.0043 | 0.0128 | 0.7360 | 0.0039 | | 84.3310 |
| rs5750642 | A | G | -0.0546 | 0.0113 | 0.517 | 0.000001357 | A | | G | -0.0104 | 0.0133 | 0.4341 | 0.0015 | | 32.4399 |
| rs60182773 | T | C | -0.163 | 0.0288 | 0.0508 | 1.431E-08 | T | | C | -0.0362 | 0.0361 | 0.3169 | 0.0026 | | 55.8882 |
| rs6074044 | T | C | 0.1634 | 0.0164 | 0.1358 | 1.79887E-23 | T | | C | -0.0589 | 0.0201 | 0.0033 | 0.0063 | | 137.2010 |
| rs6131010 | G | A | 0.4471 | 0.0118 | 0.7292 | 1E-200 | G | | A | -0.0460 | 0.0150 | 0.0022 | 0.0789 | | 1864.7860 |
| rs73112805 | G | T | 0.174 | 0.0233 | 0.062 | 7.93414E-14 | G | | T | 0.0269 | 0.0289 | 0.3519 | 0.0035 | | 76.8837 |
| rs73116741 | A | G | -0.1693 | 0.0264 | 0.0484 | 1.464E-10 | A | | G | 0.0193 | 0.0318 | 0.5443 | 0.0026 | | 57.5932 |
| rs78219644 | A | G | 0.3847 | 0.0834 | 0.0121 | 4.02004E-06 | A | | G | 0.0761 | 0.1324 | 0.5655 | 0.0035 | | 77.2487 |
| rs78549138 | C | T | -0.0887 | 0.0193 | 0.1055 | 4.40099E-06 | C | | T | -0.0146 | 0.0216 | 0.4990 | 0.0015 | | 32.3545 |
| rs79246611 | T | C | -0.1684 | 0.0326 | 0.0409 | 0.000000249 | T | | C | -0.0089 | 0.0419 | 0.8307 | 0.0022 | | 48.5118 |
|  |  |  |  |  |  |  |  | |  |  |  |  |  | |  |
| **Tumor necrosis factor receptor superfamily member 6 and severe covid-19** | | | | | | | | | | | | | | | |
|  | **Exposure** | | | | | | | **Outcome** | | | | | |  | |
| **SNP** | **Effect allele** | **Other allele** | **beta** | **se** | ***Eaf*** | ***P*-val** | **Effect allele** | | **Other allele** | **beta** | **se** | ***P*-val** | **R²** | | **F-statistic** |
| rs111693226 | T | C | -0.2492 | 0.0521 | 0.0215 | 1.70E-06 | T | | C | -0.0326 | 0.0428 | 0.4459 | 0.0026 | | 56.9955 |
| rs11202892 | T | C | 0.2383 | 0.0309 | 0.9614 | 1.22E-14 | T | | C | -0.0021 | 0.0379 | 0.9565 | 0.0042 | | 92.0837 |
| rs12359411 | A | T | 0.0694 | 0.0149 | 0.0941 | 3.08E-06 | A | | T | 0.0040 | 0.0288 | 0.8883 | 0.0008 | | 17.8795 |
| rs134177 | G | A | -0.0532 | 0.0108 | 0.7192 | 7.93E-07 | G | | A | 0.0030 | 0.0138 | 0.8262 | 0.0011 | | 24.8987 |
| rs138406468 | G | A | -0.2091 | 0.0444 | 0.0221 | 2.43E-06 | G | | A | 0.0127 | 0.0503 | 0.8012 | 0.0019 | | 41.1932 |
| rs150729260 | A | G | -0.0964 | 0.0192 | 0.0566 | 4.89E-07 | A | | G | -0.0111 | 0.0301 | 0.7114 | 0.0010 | | 21.6126 |
| rs1756775 | G | A | -0.0971 | 0.0141 | 0.1316 | 5.14E-12 | G | | A | -0.0141 | 0.0209 | 0.5016 | 0.0022 | | 46.9851 |
| rs1977081 | C | T | 0.0545 | 0.0119 | 0.187 | 4.27E-06 | C | | T | -0.0192 | 0.0226 | 0.3949 | 0.0009 | | 19.6665 |
| rs2034026 | A | T | -0.0559 | 0.0119 | 0.2354 | 2.66E-06 | A | | T | -0.0178 | 0.0216 | 0.4108 | 0.0011 | | 24.4998 |
| rs349345 | G | A | -0.0506 | 0.0107 | 0.725 | 2.36E-06 | G | | A | -0.0211 | 0.0147 | 0.1511 | 0.0010 | | 22.2343 |
| rs34967069 | T | C | 0.1419 | 0.018 | 0.116 | 3.21E-15 | T | | C | -0.0105 | 0.0313 | 0.7375 | 0.0041 | | 90.2155 |
| rs368131 | C | T | 0.0811 | 0.0174 | 0.9276 | 3.16E-06 | C | | T | 0.0149 | 0.0273 | 0.5847 | 0.0009 | | 19.2368 |
| rs4669089 | A | G | -0.0488 | 0.01 | 0.5007 | 1.20E-06 | A | | G | 0.0050 | 0.0134 | 0.7115 | 0.0012 | | 25.9361 |
| rs61954159 | T | C | -0.189 | 0.0408 | 0.0231 | 3.57E-06 | T | | C | -0.0261 | 0.0438 | 0.5513 | 0.0016 | | 35.1314 |
| rs62052447 | A | G | -0.0915 | 0.0178 | 0.0745 | 2.67E-07 | A | | G | -0.0392 | 0.0261 | 0.1333 | 0.0012 | | 25.1470 |
| rs7089946 | C | G | -0.1283 | 0.0095 | 0.4529 | 2.56E-41 | C | | G | -0.0195 | 0.0134 | 0.1438 | 0.0082 | | 178.9323 |
| rs71333623 | T | C | 0.2817 | 0.0594 | 0.0118 | 2.15E-06 | T | | C | 0.0471 | 0.0800 | 0.5557 | 0.0019 | | 40.3380 |
| rs7611124 | C | T | -0.0536 | 0.0117 | 0.1905 | 4.61E-06 | C | | T | 0.0270 | 0.0162 | 0.0952 | 0.0009 | | 19.2946 |
| rs982764 | C | T | -0.2452 | 0.01 | 0.315 | 1.52E-132 | C | | T | -0.0313 | 0.0137 | 0.0228 | 0.0259 | | 579.5196 |
|  |  |  |  |  |  |  |  | |  |  |  |  |  | |  |
| **CD40L and severe covid-19** | | | | | | | | | | | | | | | |
|  | **Exposure** | | | | | | | **Outcome** | | | | | |  | |
| **SNP** | **Effect allele** | **Other allele** | **beta** | **se** | ***Eaf*** | ***P*-val** | **Effect allele** | | **Other allele** | **beta** | **se** | ***P*-val** | **R²** | | **F-statistic** |
| rs10242069 | C | T | 0.0738 | 0.0159 | 0.1617 | 3.64502E-06 | C | | T | 0.0383 | 0.0181 | 0.0348 | 0.0015 | | 32.1716 |
| rs139710154 | A | G | -0.0732 | 0.0159 | 0.1329 | 4.35402E-06 | A | | G | -0.0223 | 0.0190 | 0.2406 | 0.0012 | | 26.9006 |
| rs1956389 | C | T | -0.0669 | 0.0144 | 0.8083 | 3.45199E-06 | C | | T | 0.0117 | 0.0168 | 0.4861 | 0.0014 | | 30.2175 |
| rs34651985 | A | G | -0.2844 | 0.0621 | 0.0112 | 0.000004561 | A | | G | -0.0124 | 0.0652 | 0.8498 | 0.0018 | | 39.0457 |
| rs4602861 | G | A | -0.0764 | 0.0135 | 0.2489 | 1.359E-08 | G | | A | 0.0145 | 0.0153 | 0.3435 | 0.0022 | | 47.5847 |
| rs57759565 | C | T | 0.0636 | 0.0136 | 0.2288 | 0.000002711 | C | | T | -0.0029 | 0.0159 | 0.8552 | 0.0014 | | 31.1005 |
| rs6532322 | G | A | -0.0547 | 0.0108 | 0.4564 | 3.99696E-07 | G | | A | 0.0299 | 0.0125 | 0.0169 | 0.0015 | | 32.3485 |
| rs72711133 | T | C | 0.0942 | 0.0196 | 0.0999 | 1.53801E-06 | T | | C | 0.0078 | 0.0224 | 0.7288 | 0.0016 | | 34.7745 |
| rs74537816 | C | T | -0.0764 | 0.0167 | 0.1365 | 0.00000488 | C | | T | 0.0305 | 0.0265 | 0.2490 | 0.0014 | | 29.9770 |
| rs7690862 | T | C | -0.2292 | 0.0461 | 0.0182 | 6.74404E-07 | T | | C | 0.1108 | 0.0497 | 0.0259 | 0.0019 | | 40.9212 |
| rs79963070 | G | A | 0.2415 | 0.0493 | 0.0179 | 9.73397E-07 | G | | A | -0.0264 | 0.0678 | 0.6970 | 0.0021 | | 44.7037 |

Table S3. Mendelian randomization estimates for the association of TNFs with severe COVID-19.

|  | **Multiplicative random-effect IVW** | | | | | | **Weighted median** | | | | | | **MR Egger** | | | | | |
| --- | --- | --- | --- | --- | --- | --- | --- | --- | --- | --- | --- | --- | --- | --- | --- | --- | --- | --- |
| **Phenotype** | **beta** | **se** | ***P*-val** | **OR** | **95% CI lower bound** | **95% CI upper bound** | **beta** | **se** | ***P*-val** | **OR** | **95% CI lower bound** | **95% CI upper bound** | **beta** | **se** | ***P*-val** | **OR** | **95% CI lower bound** | **95% CI upper bound** |
| TRANCE | -0.018 | 0.038 | 0.643 | 0.982 | 0.911 | 1.059 | -0.050 | 0.054 | 0.360 | 0.952 | 0.856 | 1.058 | 0.025 | 0.073 | 0.739 | 1.025 | 0.888 | 1.183 |
| TRAIL | 0.004 | 0.030 | 0.902 | 1.004 | 0.946 | 1.064 | 0.005 | 0.042 | 0.912 | 1.005 | 0.930 | 1.085 | -0.046 | 0.048 | 0.346 | 0.955 | 0.870 | 1.049 |
| TRAIL-R2 | -0.131 | 0.072 | 0.070 | 0.877 | 0.762 | 1.011 | -0.125 | 0.083 | 0.130 | 0.882 | 0.745 | 1.045 | -0.019 | 0.119 | 0.877 | 0.981 | 0.776 | 1.240 |
| TNFSF14 | 0.013 | 0.033 | 0.698 | 1.013 | 0.950 | 1.080 | 0.049 | 0.041 | 0.234 | 1.050 | 0.969 | 1.139 | 0.039 | 0.047 | 0.424 | 1.039 | 0.948 | 1.140 |
| TNF-R1 | -0.100 | 0.070 | 0.152 | 0.905 | 0.789 | 1.038 | -0.049 | 0.092 | 0.598 | 0.953 | 0.797 | 1.139 | 0.048 | 0.115 | 0.681 | 1.050 | 0.837 | 1.316 |
| TNF-R2 | -0.065 | 0.048 | 0.175 | 0.937 | 0.853 | 1.029 | -0.050 | 0.073 | 0.499 | 0.952 | 0.824 | 1.099 | 0.011 | 0.084 | 0.902 | 1.011 | 0.857 | 1.192 |
| CD40 | -0.085 | 0.027 | **0.002** | 0.918 | 0.871 | 0.969 | -0.098 | 0.032 | **0.002** | 0.906 | 0.850 | 0.966 | -0.111 | 0.040 | **0.012** | 0.895 | 0.827 | 0.968 |
| FAS | 0.093 | 0.042 | **0.026** | 1.098 | 1.011 | 1.191 | 0.127 | 0.051 | **0.014** | 1.135 | 1.025 | 1.258 | 0.151 | 0.070 | **0.049** | 1.163 | 1.014 | 1.334 |
| CD40L | -0.098 | 0.098 | 0.316 | 0.907 | 0.749 | 1.098 | -0.111 | 0.113 | 0.326 | 0.895 | 0.726 | 1.105 | -0.105 | 0.256 | 0.693 | 0.901 | 0.545 | 1.488 |

Table S4. Assessment of the presence of horizontal pleiotropy (MR-Egger intercept).

|  |  |  | **MR-Egger intercept** | | |
| --- | --- | --- | --- | --- | --- |
| **Phenotype** | **Description** | **# of instrument SNP** | **Estimate** | **SE** | ***P*** |
| TRANCE | TNF-related activation-induced cytokine | 18 | -0.005 | 0.008 | 0.503 |
| TRAIL | TNF-related apoptosis-inducing ligand | 23 | 0.009 | 0.007 | 0.199 |
| TRAILR2 | TNF-related apoptosis-inducing ligand receptor 2 | 14 | -0.013 | 0.011 | 0.266 |
| TNFSF14 | Tumor necrosis factor ligand superfamily member 14 | 18 | -0.006 | 0.008 | 0.455 |
| TNFR1 | Tumor necrosis factor receptor 1 | 15 | -0.016 | 0.010 | 0.138 |
| TNFR2 | Tumor necrosis factor receptor 2 | 26 | -0.007 | 0.007 | 0.289 |
| CD40 | Tumor necrosis factor receptor superfamily member 5 | 22 | 0.005 | 0.006 | 0.388 |
| FAS | Tumor necrosis factor receptor superfamily member 6 | 16 | -0.009 | 0.009 | 0.322 |
| CD40L | CD40 ligand | 11 | 0.001 | 0.021 | 0.978 |

Table S5. Mendelian randomization estimates for the association of COVID-19 with exposures.

| **Exposure** | **Outcome** | **Method** | **# of instrument SNP** | **b** | **se** | **OR** | **95% CI lower bound** | **95% CI upper bound** | ***P*-val** |
| --- | --- | --- | --- | --- | --- | --- | --- | --- | --- |
| severe COVID-19 | TNF-related activation-induced cytokine levels | IVW | 31 | 0.024 | 0.017 | 0.159 | 0.991 | 1.024 | 1.059 |
| severe COVID-19 | TNF-related apoptosis-inducing ligand levels | IVW | 31 | 0.019 | 0.020 | 1.019 | 0.980 | 1.060 | 0.342 |
| severe COVID-19 | TNF-related apoptosis-inducing ligand receptor 2 levels | IVW | 31 | -0.004 | 0.021 | 0.996 | 0.956 | 1.037 | 0.845 |
| severe COVID-19 | Tumor necrosis factor ligand superfamily member 14 levels | IVW | 31 | 0.004 | 0.017 | 1.004 | 0.970 | 1.038 | 0.822 |
| severe COVID-19 | Tumor necrosis factor receptor 1 levels | IVW | 31 | -0.003 | 0.018 | 0.997 | 0.963 | 1.032 | 0.851 |
| severe COVID-19 | Tumor necrosis factor receptor 2 levels | IVW | 31 | -0.016 | 0.018 | 0.360 | 0.984 | 0.950 | 1.019 |
| severe COVID-19 | Tumor necrosis factor receptor superfamily member 5 levels | IVW | 31 | -0.012 | 0.017 | 0.988 | 0.956 | 1.021 | 0.476 |
| severe COVID-19 | Tumor necrosis factor receptor superfamily member 6 levels | IVW | 31 | 0.014 | 0.016 | 1.014 | 0.983 | 1.046 | 0.381 |
| severe COVID-19 | CD40L | IVW | 31 | -0.018 | 0.016 | 0.982 | 0.952 | 1.013 | 0.246 |

**
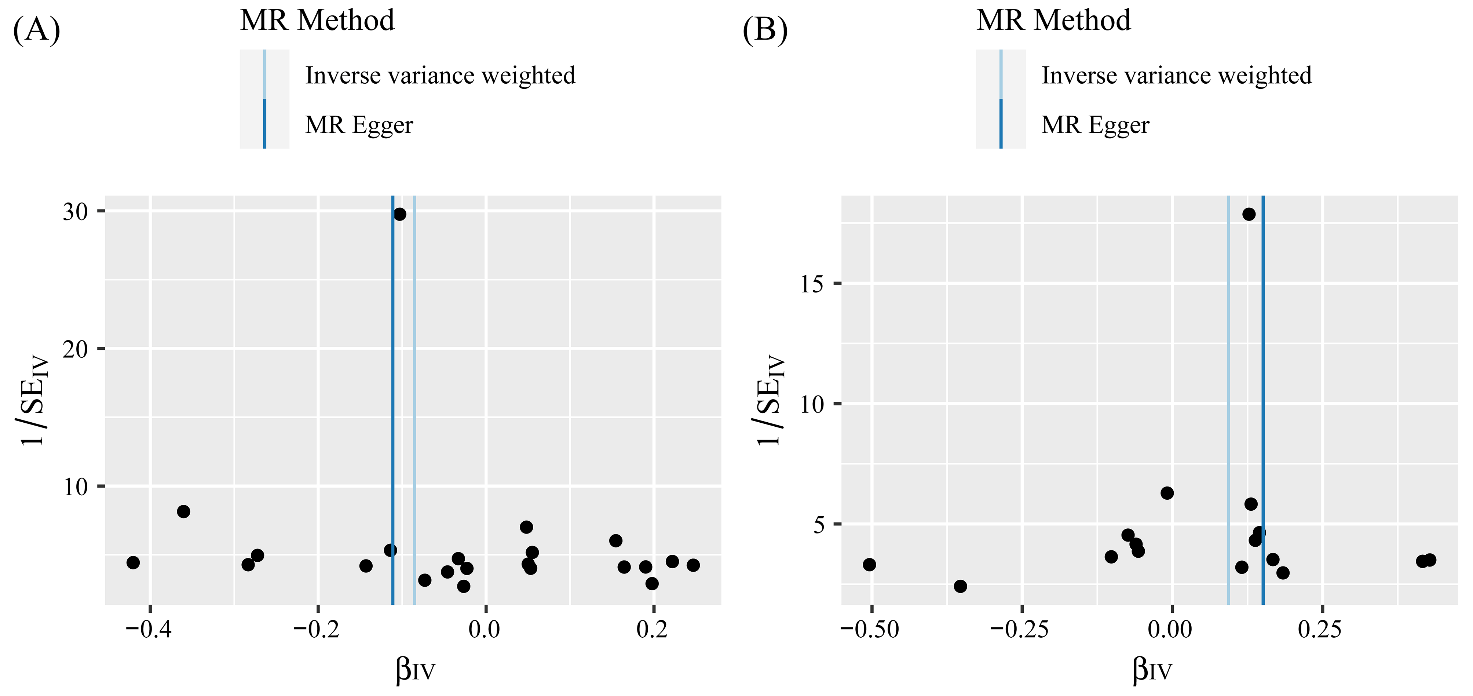
**

Figure S1. Funnel plot of SNPs associated with CD40 levels (A) and FAS levels (B) and severe COVID-19 after outliers removal with MR-PRESSO.

**
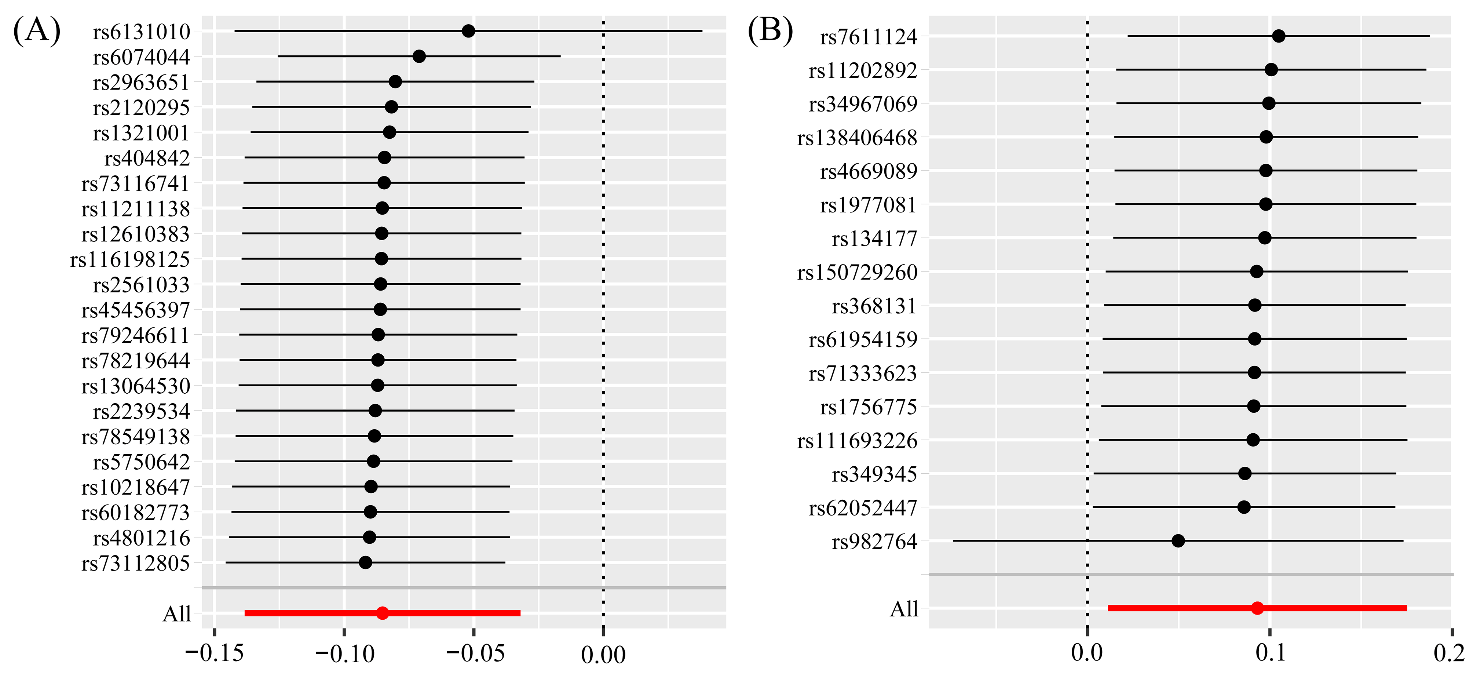
**

Figure S2. Plots of “leave-one-out” analyses for MR analyses of the causal effect of CD40 levels (A) and FAS levels (B) on severe COVID-19.
